# Duration of COVID-19 symptoms in children: a longitudinal study in a Rio de Janeiro favela, Brazil

**DOI:** 10.1136/bmjopen-2024-095622

**Published:** 2025-07-06

**Authors:** Fernanda Esthefane Garrides Oliveira, Leonardo Bastos, Raquel de Vasconcellos Carvalhães de Oliveira, Heloísa Ferreira Pinto Santos, Luana Santana Damasceno, Luiza Sales Franco, Liege Maria Abreu de Carvalho, Trevon Louis Fuller, Lusiele Guaraldo, Marilia Carvalho, Patrícia Brasil

**Affiliations:** 1 Oswaldo Cruz Institute, Oswaldo Cruz Foundation, Rio de Janeiro, Brazil; 2 Scientific Computing Program, Oswaldo Cruz Foundation, Rio de Janeiro, Brazil; 3 Evandro Chagas National Institute of Infectious Disease, Oswaldo Cruz Foundation, Rio de Janeiro, Brazil; 4 University of California Los Angeles, Los Angeles, California, USA

**Keywords:** COVID-19, Public health, Community child health, Paediatric infectious disease & immunisation

## Abstract

**Abstract:**

**Objectives:**

COVID-19 in children is generally of short duration, but some may take longer to recover. This study investigated the time to symptom resolution following SARS-CoV-2 infection among children in a community setting on the outskirts of an urban centre in Brazil.

**Design:**

Prospective cohort study.

**Setting:**

This is a community-based cohort of children living in Manguinhos, a favela in Rio de Janeiro. The cohort was followed through home visits and telephone monitoring of symptoms. The analysis focused on symptomatic children from this cohort with confirmed SARS-CoV-2 infection. Recovery time was defined as the interval between the first date with symptoms and the first date without symptoms following a positive SARS-CoV-2 test.

**Participants:**

A total of 1276 children (boys and girls aged 2–<14 years) were recruited between 2020 and 2022, with 253 testing positive for SARS-CoV-2. The inclusion criterion was SARS-CoV-2-positive children, while the exclusion criteria were loss to follow-up and asymptomatic cases during the acute phase of COVID-19.

**Outcome measure:**

COVID-19 recovery time, assessed based on change points on the symptom persistence probability curve (Kaplan-Meier).

**Results:**

Among children who tested positive, 148 (60%) were symptomatic. The median recovery time was 11 days (IQR: 7–16). Two inflection points were identified on the Kaplan-Meier curve: days 16 and 34. Children who were ill during the Omicron wave took longer to recover. More boys became asymptomatic within the first 15 days; about 93% of girls recovered by day 33, and boys were more common among those who recovered in ≥34 days. Children aged 6–<14 years often recovered within 2 weeks; however, both this group and the 2–<6 years group constituted the majority of those with delayed recovery.

**Conclusions:**

Among children from a vulnerable area in Rio de Janeiro, recovery time was longer than that reported in other countries, with 9.5% of children experiencing persistent symptoms for more than 33 days. These findings are crucial for understanding the implications of COVID-19 in specific socioeconomic contexts and the dynamics of paediatric recovery in community settings.

**STRENGTHS AND LIMITATIONS OF THIS STUDY**

- The prospective design and the continuous symptom monitoring (through home visits and phone calls to children’s guardians) minimise information bias.
- The analytical approach used to identify change points on the symptom persistence probability curve helps distinguish between the acute phase of the disease, where recovery is more likely, and the chronic phase, where the probability of becoming asymptomatic is more stable yet lower.
- The size of the group of children who had symptomatic COVID-19 during the acute phase limits the depth of analyses.
- The high proportion of unvaccinated children at the time of COVID-19 infection limits analysis related to vaccination.

## Introduction

 As of August 2024, over 776 million confirmed cases of COVID-19 have been reported worldwide, significantly affecting the health of individuals both in the short and long term. People affected by the disease may develop mild symptoms that resolve within a few days or weeks, or may experience longer-lasting effects that persist, develop or fluctuate after infection.^1 2^

Due to the milder course of the disease in children compared with adults, research on persisting symptoms following COVID-19 in this age group has been relatively overlooked. This gap in knowledge could hinder our ability to identify and address potential long-term health problems in children.^3^

Characterising the clinical presentation and course of COVID-19 in children and understanding the duration of symptoms and their potential evolution over time are priorities for research involving post-COVID-19 syndrome in children.^1^ Furthermore, investigating long covid in children in low-income and middle-income countries, especially in vulnerable areas, can illuminate the pandemic’s disproportionate impact on disadvantaged communities. This could help identify risk factors and health disparities that exacerbate symptoms and hinder recovery.

In this study, we evaluated symptomatic children (<14 years old) with a positive real-time reverse transcriptase-polymerase chain reaction (RT-PCR) test for SARS-CoV-2 in a cohort of children residing in Manguinhos, a favela in Rio de Janeiro. To assess the time to resolution of symptoms from the acute phase of SARS-CoV-2 infection, the first 9 months of follow-up after the date of a positive test were considered.

## Methods

### Study setting

Manguinhos is a neighbourhood in the North Zone of Rio de Janeiro, Brazil, also known as the Manguinhos Complex due to the presence of multiple favelas (slums). The Brazilian Institute of Geography and Statistics (in Portuguese *Instituto Brasileiro de Geografia e Estatística*) defines favelas and urban communities as popular territories that have emerged through various strategies used by the population to autonomously and collectively meet their housing needs, given the insufficiency and inadequacy of public policies and investments aimed at ensuring the right to the city.^4^ These territories reflect the sociospatial inequalities of Brazilian urbanisation, characterised by a predominance of households with insecure land tenure, a lack or precarious provision of public services, predominantly self-built housing and infrastructure that often do not conform to public regulations, and/or locations in areas with occupation restrictions or environmental risk.^4^

In the 2010 Brazilian Demographic Census, Manguinhos had an estimated population of 36 160 inhabitants, of whom 9686 were children aged 0–<14 years (27% of the population).^5^ Most households had three or more residents (66.5%),^6^ and the population was predominantly black (64%).^7^ The local population has declined in recent years, with the most recent Brazilian Demographic Census (2022) estimating it at 28 855 inhabitants, although detailed age distribution data are not yet available.^8^

Manguinhos is characterised by one of the lowest Human Development Index scores in Rio de Janeiro, among the lowest levels of per capita household income,^9^ high rates of urban violence, and environmental health issues such as flooding, sewage discharged into canals and contamination of water used for consumption.^10 11^ A local survey covering 955 households found that, among adults aged 25 years or older, 66% of men and 69% of women had between 0 and 8 years of education, and 22% of young people aged 14–19 were engaged in some form of work.^12^ Among the households surveyed, 35% had experienced food insecurity in the previous 3 months, only 48% had regular rubbish collection and 9% of residents had asthma or bronchitis; additionally, among adults aged 30 or older, 36% had hypertension, 9% had diabetes, approximately 20% were smokers and 12% consumed alcohol three or more times per week.^12^

### Design, study population and data collection

The Manguinhos Family Cohort is a prospective, open and dynamic cohort study without a formal sampling process, where any child attending the health centre during the study period was eligible for participation. The cohort was recruited at the Germano Sinval Faria Municipal Health Centre, affiliated with the Sergio Arouca National School of Public Health (Oswaldo Cruz Foundation). This health centre is part of the Brazilian public health system and is responsible for primary healthcare in the Manguinhos territory. All children who visited the health centre between May 2020 and May 2022, whether for elective consultation, immunisation, emergency care or accompanying a family member, were eligible to participate in the study, regardless of whether they were symptomatic or not.

At enrolment, a questionnaire was administered to the child’s parents or guardians to collect data on medical history, clinical and demographic factors and COVID-19 vaccination status. If the child was symptomatic, an additional clinical questionnaire was completed to record symptoms. The child’s home address and the contact details of the guardians were obtained, and this information was used to schedule home visits and include other children residing in the same household. Parents or adult guardians who refused to participate, who stated they had no time to participate or children whose parents or legal guardians were not present at the time of the home visit, preventing consent, were not included in the study.^13^ All eligible families, that is, those whose children visited the health centre between May 2020 and May 2022, were approached during this period. This corresponded to approximately 1100 families, of whom around 77% agreed to participate in the research. Children and their household contacts could remain in follow-up for up to 2 years.^13^

The study protocol stipulated that home visits (for sample collection) and follow-up phone calls would be carried out regardless of whether the child was symptomatic or had a positive test. Active home visits and phone calls were also strategies to retain families in the cohort, as well as having fieldworkers who lived in the community facilitate rapport with participants. Visits (for sample collection) were planned for days 1 (enrolment in the cohort), 14 and 28 after enrolment, followed by once every 3 months in the first year and twice in the second year. Phone calls were scheduled to take place within 10 days of enrolment; then between 11 and 17 days, 28 and 32 days, and 42 and 45 days; then at 2, 3, 4, 6, 9 and 12 months; and then between 15 and 18 months and between 21 and 24 months. When the child was symptomatic, phone calls became more frequent, occurring at least once a week until symptom resolution. Further details on the cohort can be found in a previous publication.^13^ To assess symptom resolution, the eligibility criteria for the present analysis were that the child had a confirmed SARS-CoV-2 infection and was symptomatic during the acute phase of COVID-19.

### Measures

Information on sex (male/female) and age (categorised into age groups: <2 years, 2–<6 years and 6–<14 years) was assessed. During the recruitment interview, families reported on bronchitis/asthma, rhinitis, congenital infections and other comorbidities (this was an open-ended question that allowed families to report any additional conditions). These data categorised pre-existing comorbidities as ‘yes’ or ‘no/unknown’.

Vaccination status for COVID-19 at the time of a positive RT-PCR for SARS-CoV-2 was defined as ‘not vaccinated’ or ‘vaccination initiated’, based on information collected regarding vaccination (number of doses, manufacturer and date).

Nasopharyngeal swabs were tested by real-time RT-PCR to amplify the E gene and the RdRp region of the Orf1ab gene of SARS-CoV-2 using the molecular SARS-CoV-2 kit from Bio-Manguinhos. Results with cycle thresholds ≤40 (E target) and ≤35 (Rp target) were considered positive.^14^ SARS-CoV-2 infection was defined as a positive RT-PCR test.

Clinical manifestations evaluated in children included cough, sneezing, nasal congestion/runny nose, sore throat, fever, chills, weakness/prostration (fatigue), difficulty breathing, headache, abdominal pain, diarrhoea, nausea, vomiting, conjunctivitis, body rash, arthralgia, seizures, anosmia and dysgeusia. If at least one of these symptoms was reported at the time of the interview/follow-up contact, the child was then considered symptomatic.

The variable ‘variant in circulation at the time of RT-PCR’ was used to represent the predominant COVID-19 variant circulating in Rio de Janeiro at the time the child’s test was positive. This variable was categorised as ‘prior to variants of concern’ for the period up to January 2021, ‘Gamma/Delta’ for the period from late January 2021 to November 2021 and ‘Omicron’ for the period from December 2021 onwards. The period of predominance for each variant was defined based on genomic surveillance data from the state of Rio de Janeiro, systematised by the Oswaldo Cruz Foundation Genomic Network.^15^

### Data analysis

For the present analysis, the date of the positive RT-PCR test for SARS-CoV-2 (laboratory diagnosis) was considered the start of follow-up. The follow-up period to assess the children’s recovery was defined as 9 months after the positive test due to the difficulty in maintaining periodic contact after this period but also due to interest in recovery following the initial infection. As this is a paediatric population, after a long period, children may again experience symptoms due to common health issues in this age group.

Children who were symptomatic during the acute phase of the disease were defined as those who presented any evaluated symptom on the test date or within up to 10 days from the test date. The event was the resolution of symptoms (when the child became asymptomatic). Children who remained symptomatic until the end of the follow-up period were censored at that date and those lost to follow-up were censored at the date of the last recorded contact/visit. The interval between the first reported date of symptoms and the first subsequent contact date when the child was asymptomatic, within 9 months from the positive COVID-19 test result, was considered the time to event.

Kaplan-Meier analysis was used to estimate the survival curve for symptom persistence and to investigate change points. The definition of a change point in the hazard function of a survival distribution assumes a constant hazard rate after the change point and a decreasing hazard rate before the change point.^16^ For its estimation, time is divided into equidistant intervals, and exact binomial tests are performed for each interval. The change point is estimated by fitting a two-phase piecewise constant regression model (a so-called stump) to the p values as the outcome variable.^16^ Subsequently, from the identified change points, absolute and relative frequencies are used to describe the characteristics of the children according to the period of symptom resolution. To assess differences in the distribution of groups, a variation indicator (Δ) was shown, corresponding to the difference between percentages considering a reference category, that is, percentage points (pp). Finally, the proportion of each symptom reported by children over time was estimated according to their symptom resolution period. Information was provided on children who had the longest persistence of symptoms. Although 95% CIs are provided for the proportion estimates, the focus of our analysis is on the differences in point estimates. This is due to the limited sample size, which affects the precision of the CIs, resulting in a broader range that may not capture the true variability of the estimates.

All analyses were conducted using R V.4.3.2 software (The R Foundation for Statistical Computing, Vienna, Austria, 2023; http://www.r-project.org), with the packages CPSurv V.1.0.0,^17^ Survival V.3.5-7^18^ and epiR V.2.0.65.^19^

### Patient and public involvement

None.

## Results

**Figure 1:**
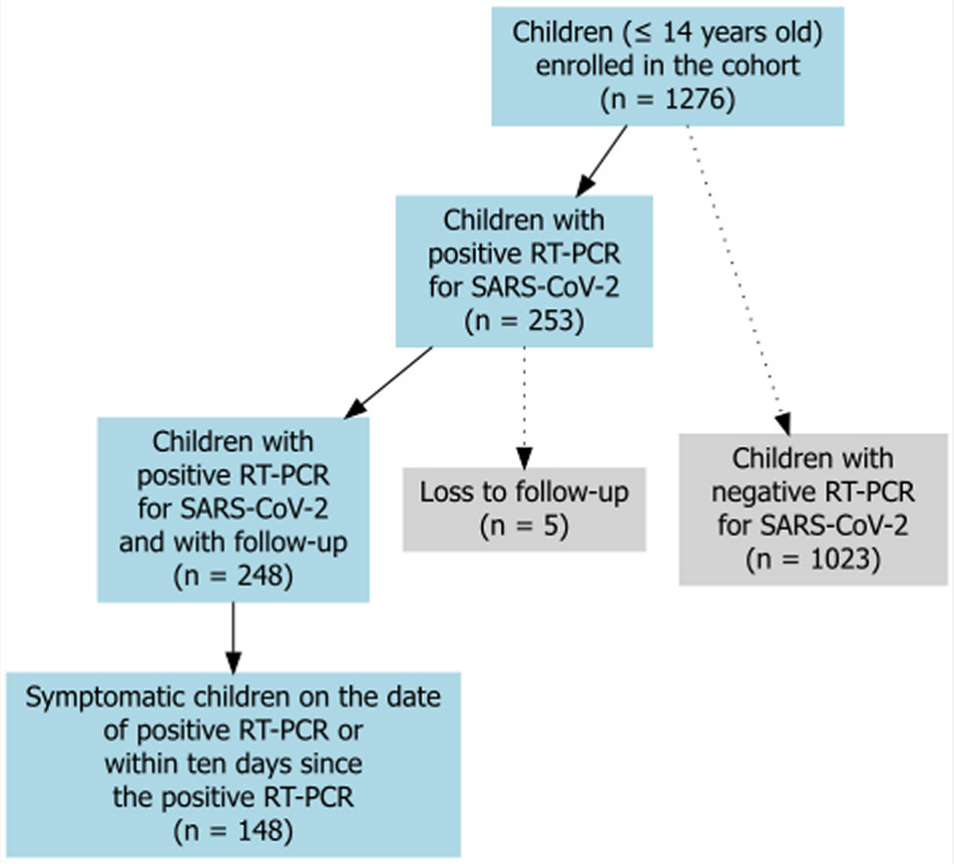
Flow chart of children enrolled in the cohort and the number of symptomatic children at the time of positive real-time reverse transcriptase-polymerase chain reaction (RT-PCR) for SARS-CoV-2, Manguinhos Family Cohort, 2020–2023.

A total of 852 families were included in the study, with 1276 children under the age of 14 enrolled, of whom 253 (19.83%) tested positive for SARS-CoV-2 by RT-PCR (figure 1), and 248 of them remained under follow-up (98.02%). Of these, 148 children (59.68%) exhibited symptoms on the date of RT-PCR (135/148, 91.22%) or within 10 days of testing (13/148, 8.78%).

**Figure 2:**
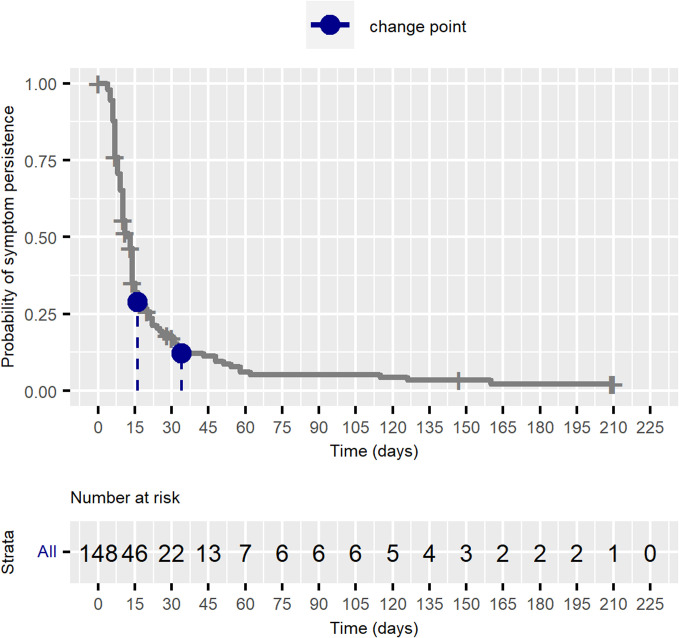
Kaplan-Meier curve for the probability of symptom persistence among symptomatic children at the time of positive real-time reverse transcriptase-polymerase chain reaction (RT-PCR) for SARS-CoV-2, Manguinhos Family Cohort, 2020–2023.

Of the 148 children with acute-phase symptoms who were followed up, 134 experienced symptom resolution, while 14 remained symptomatic at the end of the follow-up period. Among the 134 children who became asymptomatic, the mean time to symptom resolution was 17.5 days (95% CI 13.84 to 21.16), with a median of 11 days (first quartile on day 7 and third quartile on day 16). Figure 2 shows the Kaplan-Meier curve for the probability of symptoms persisting beyond any specified time. Most children became asymptomatic within the first 2 weeks after the symptom onset, so that by the 13th day the probability of symptoms persisting was 0.50. The curve exhibited an inflection: an initial rapid decline, with many children becoming symptom-free in the early days, and a slower decline over time, reflecting the children for whom symptoms persisted for several months. Change point investigation within 30 days indicated an inflection point on the curve on the 16th day, while investigation for a change point after 30 days indicated an inflection point on the 34th day.

Table 1 provides an overall description of the children’s profile concerning the period of symptom resolution. The group of symptomatic children was primarily composed of male children (80/148, 54.05%), children aged 6–<14 years (75/148, 50.68%), children who were not vaccinated for COVID-19 (138/148, 93.24%) and children without pre-existing comorbidities (100/148, 67.57%). Children who tested positive during the Gamma/Delta period were slightly more frequent (59/148, 39.86%). Among the 48 children with pre-existing comorbidities, 17 had rhinitis, 17 had bronchitis/asthma, 5 had both rhinitis and bronchitis/asthma, 4 had one of these conditions along with another condition and 5 had a single condition different from these, with many of the additional conditions involving some form of disability, such as autism. During the Omicron circulation period, children were less likely to experience symptom resolution within the first 15 days (17.27 pp lower) compared with the period prior to variants of concern. However, they were more likely to experience symptom resolution between days 16 and 33 (12.98 pp higher) or after 34 days (4.29 pp higher) during the Omicron period.

**Table 1:** Characterisation of 148 symptomatic children at the time of positive real-time reverse transcriptase-polymerase chain reaction (RT-PCR) for SARS-CoV-2, total group and concerning the period of symptom resolution, Manguinhos Family Cohort, 2020–2023

	Total	Time to symptom resolution/censorship					
		≤15th day		16th–≤33rd day		≥34th day	
	n (%) (95% CI)	n (%) (95% CI)	Δ	n (%) (95% CI)	Δ	n (%) (95% CI)	Δ
Total	148 (100.00)	106 (71.62) (63.64 to 78.72)		28 (18.92) (12.96 to 26.17)		14 (9.46) (5.27 to 15.36)	
Variant in circulation at the time of PCR							
Prior to variants of concern	44 (29.73) (22.50 to 37.79)	34 (77.27) (62.16 to 88.53)	Ref	7 (15.91) (6.64 to 30.07)	Ref	3 (6.82) (1.43 to 18.66)	Ref
Gamma/Delta	59 (39.86) (31.92 to 48.23)	45 (76.27) (63.41 to 86.38)	−1.00	8 (13.56) (6.04 to 24.98)	−2.35	6 (10.17) (3.82 to 20.83)	+3.35
Omicron	45 (30.41) (23.12 to 38.50)	27 (60.00) (44.33 to 74.30)	−17.27	13 (28.89) (16.37 to 44.32)	+12.98	5 (11.11) (3.71 to 24.05)	+4.29
Sex							
Male	80 (54.05) (45.68 to 62.27)	59 (73.75) (62.72 to 82.96)	Ref	12 (15.00) (8.00 to 24.74)	Ref	9 (11.25) (5.28 to 20.29)	Ref
Female	68 (45.95) (37.73 to 54.32)	47 (69.12) (56.74 to 79.76)	−4.63	16 (23.53) (14.09 to 35.38)	+8.00	5 (7.35) (2.43 to 16.33)	−3.90
Age group (years)							
<2	31 (20.95) (14.70 to 28.40)	20 (64.52) (45.37 to 80.77)	Ref	8 (25.81) (11.86 to 44.61)	Ref	3 (9.68) (2.04 to 25.75)	Ref
2–<6	42 (28.38) (21.28 to 36.37)	26 (61.90) (45.34 to 76.43)	−2.62	10 (23.81) (12.05 to 39.45)	−2.00	6 (14.29) (5.43 to 28.54)	+4.61
6–<14	75 (50.68) (42.34 to 58.98)	60 (80.00) (69.17 to 88.35)	+15.48	10 (13.33) (6.58 to 23.16)	−12.48	5 (6.67) (2.20 to 14.88)	−3.01
Vaccination status at the time of PCR							
Vaccination initiated	10 (6.76) (3.29 to 12.08)	6 (60.00) (26.24 to 87.45)	Ref	1 (10.00) (0.25 to 44.50)	Ref	3 (30.00) (6.67 to 65.25)	Ref
Not vaccinated	138 (93.24) (87.93 to 96.71)	100 (72.46) (64.22 to 79.72)	+12.46	27 (19.57) (13.31 to 27.18)	+9.57	11 (7.97) (4.05 to 13.82)	−22.03
Pre-existing comorbidity							
No/unknown	100 (67.57) (59.39 to 75.02)	73 (73.00) (63.20 to 81.39)	Ref	19 (19.00) (11.84 to 28.07)	Ref	8 (8.00) (3.52 to 15.16)	Ref
Yes	48 (32.43) (24.98 to 40.61)	33 (68.75) (53.75 to 81.34)	−4.25	9 (18.75) (8.95 to 32.63)	−0.25	6 (12.50) (4.73 to 25.25)	+4.50

’Δ’ corresponds to the variation in point estimates between the percentages of the category and the reference category, expressed as percentage points. The ‘total’ column sums to 100% in the column, and the other columns sum to 100% across the row.

Ref, reference category.

Male children were more likely to experience a rapid recovery from COVID-19 symptoms within 15 days (59/80, 73.75%), but were also more prone to prolonged illness, taking over 33 days to recover (9/80 (11.25%) vs 5/68 (7.35%) for female children). Children aged 6–14 exhibited the quickest recovery from COVID-19, with the highest percentage of symptoms resolving within the first 15 days (60/75, 80%). However, despite the faster initial recovery, both this group and the 2–<6 years age group had a similar number of children who took longer to recover, with symptom resolution occurring after 33 days. Few children had been vaccinated for COVID-19 when they tested positive for SARS-CoV-2 by RT-PCR (10/148, 6.76%), and among them 60% recovered from their symptoms within 15 days (table 1). Children without pre-existing conditions were more likely to recover from COVID-19 symptoms within 15 days, with a 4.25% higher recovery rate.

The most frequent symptoms reported were cough (92/148, 62.16%), sneezing (87/148, 58.78%), nasal congestion/runny nose (86/148, 58.11%) and fever (77/148, 52.03%) (table 2). Meanwhile, the least common symptoms were body rash (8/148, 5.41%) and arthralgia (7/148, 4.73%). Among the 19 symptoms, seizures were not observed in any child. The largest differences observed in symptom reporting were in the comparison of point estimates for reports of sore throat and sneezing. In both cases, children with symptom resolution after 33 days more frequently reported these symptoms, while children with symptom resolution between 16 and 33 days reported them less frequently, with a difference of 25 pp for sneezing and 32.15 pp for sore throat between these groups. Weakness/prostration (fatigue) was reported by 32.08% (34/106) of children within the group that recovered faster and by 14.29% (2/14) of children who recovered after 33 days. Conversely, fever and nasal congestion/runny nose were more frequently reported in the group that took 16–33 days to recover from their symptoms.

**Table 2:** Symptom reports within each Time to Symptom Resolution/Censorship group, symptomatic children at the time of positive real-time reverse transcriptase-polymerase chain reaction (RT-PCR) for SARS-CoV-2, Manguinhos Family Cohort, 2020–2023

Reported symptoms	Total	Time to symptom resolution/censorship		
		≤15th day	16th–≤33rd day	≥34th day
	% (95% CI)	% (95% CI)	% (95% CI)	% (95% CI)
Cough	62.16 (53.83 to 69.99)	64.15 (54.26 to 73.23)	57.14 (37.18 to 75.54)	57.14 (28.86 to 82.34)
Sneezing	58.78 (50.41 to 66.80)	60.38 (50.41 to 69.75)	46.43 (27.51 to 66.13)	71.43 (41.90 to 91.61)
Nasal congestion/runny nose	58.11 (49.73 to 66.16)	54.72 (44.75 to 64.41)	71.43 (51.33 to 86.78)	57.14 (28.86 to 82.34)
Fever	52.03 (43.67 to 60.30)	50.94 (41.05 to 60.78)	60.71 (40.58 to 78.50)	42.86 (17.66 to 71.14)
Headache	42.57 (34.49 to 50.95)	44.34 (34.69 to 54.31)	35.71 (18.64 to 55.94)	42.86 (17.66 to 71.14)
Weakness/prostration (fatigue)	29.05 (21.89 to 37.08)	32.08 (23.34 to 41.84)	25.00 (10.69 to 44.87)	14.29 (1.78 to 42.81)
Sore throat	22.97 (16.47 to 30.60)	23.59 (15.88 to 32.82)	10.71 (2.27 to 28.23)	42.86 (17.66 to 71.14)
Diarrhoea	18.92 (12.96 to 26.17)	15.09 (8.88 to 23.35)	28.57 (13.22 to 48.67)	28.57 (8.39 to 58.10)
Abdominal pain	18.92 (12.96 to 26.17)	17.93 (11.15 to 26.57)	21.43 (8.30 to 40.95)	21.43 (4.66 to 50.80)
Difficulty breathing	17.57 (11.81 to 24.67)	20.76 (13.49 to 29.72)	10.71 (2.27 to 28.23)	7.14 (0.18 to 33.87)
Anosmia	11.49 (6.84 to 17.75)	13.21 (7.41 to 21.17)	7.14 (0.88 to 23.50)	7.14 (0.18 to 33.87)
Vomiting	10.81 (6.31 to 16.96)	9.43 (4.62 to 16.67)	10.71 (2.27 to 28.23)	21.43 (4.66 to 50.80)
Nausea	10.14 (5.78 to 16.17)	8.49 (3.96 to 15.51)	14.29 (4.03 to 32.67)	14.29 (1.78 to 42.81)
Chills	8.78 (4.76 to 14.55)	8.49 (3.96 to 15.51)	7.14 (0.88 to 23.50)	14.29 (1.78 to 42.81)
Dysgeusia	8.11 (4.26 to 13.74)	10.38 (5.30 to 17.81)	3.57 (0.09 to 18.35)	No report
Conjunctivitis	6.76 (3.29 to 12.08)	7.55 (3.32 to 14.33)	7.14 (0.88 to 23.50)	No report
Body rash	5.41 (2.36 to 10.37)	6.60 (2.70 to 13.13)	3.57 (0.09 to 18.35)	No report
Arthralgia	4.73 (1.92 to 9.50)	5.66 (2.11 to 11.91)	No report	7.14 (0.18 to 33.87)

Table 3 presents the group of 14 children with prolonged symptom duration. This group included a higher proportion of children who tested positive during the Gamma/Delta circulation period (6/14, 42.86%), boys (9/14, 64.29%), children aged 2–<6 years (6/14, 42.86%) and children without pre-existing comorbidities (8/14, 57.14%). Additionally, there were two cases each of boys aged 6–<14 years with a positive test during the Omicron period, boys aged <2 years with a positive test during the Gamma/Delta period, boys aged 2–<6 years with a positive test before variants of concern and girls aged 2–<6 years with a positive test during the Omicron period. Notably, the highest number of symptoms was reported by a boy in the 6–<14 years age group with a positive test during the Omicron period (11 symptoms) and a girl in the 2–<6 years age group with a positive test during the same period (14 symptoms), both without pre-existing comorbidities.

**Table 3:** Characterisation of children with symptom resolution after 33 days from the onset of symptoms, Manguinhos Family Cohort, 2020–2023

Child	Variant in circulation at the time of PCR	Sex	Age group (years)	Pre-existing comorbidities	Reported symptoms
1	Prior to variants of concern	Male	2–<6	No/unknown	Headache
2	Prior to variants of concern	Male	2–<6	No/unknown	Cough
3	Prior to variants of concern	Female	6–<14	Bronchitis/asthma, rhinitis	Nasal congestion/runny nose, sneezing, headache, anosmia
4	Gamma/Delta	Male	6–<14	Rhinitis, diabetes	Sore throat
5	Gamma/Delta	Male	2–<6	Rhinitis	Cough
6	Gamma/Delta	Male	<2	G6PD deficiency	Sneezing, cough
7	Gamma/Delta	Female	6–<14	No/unknown	Sore throat, nasal congestion/runny nose, sneezing, fever, headache
8	Gamma/Delta	Male	<2	No/unknown	Nasal congestion/runny nose, sneezing, cough, fever, diarrhoea
9	Gamma/Delta	Female	2–<6	No/unknown	Sore throat, nasal congestion/runny nose, sneezing, fever, vomiting, abdominal pain, diarrhoea, weakness/prostration (fatigue)
10	Omicron	Female	2–<6	No/unknown	Sneezing
11	Omicron	Male	<2	Bronchitis/asthma	Nasal congestion/runny nose, sneezing, cough
12	Omicron	Male	6–<14	Bronchitis/asthma, rhinitis, sinusitis	Sore throat, nasal congestion/runny nose, sneezing, cough, fever, headache
13	Omicron	Male	6–<14	No/unknown	Sore throat, nasal congestion/runny nose, sneezing, cough, fever, chills, headache, vomiting, nausea, abdominal pain, diarrhoea
14	Omicron	Female	2–<6	No/unknown	Sore throat, nasal congestion/runny nose, sneezing, difficulty breathing, cough, fever, chills, headache, vomiting, nausea, abdominal pain, diarrhoea, weakness/prostration (fatigue), arthralgia

G6PD, glucose-6-phosphate dehydrogenase.

## Discussion

Our findings demonstrate that symptomatic SARS-CoV-2 infection in children under 14 years of age residing in a favela territory in Rio de Janeiro is typically short-lived, with more than half of the children becoming asymptomatic within 15 days. However, prolonged illness may occur, particularly among children who tested positive during the Omicron circulation period, preschool-age children, boys and those with pre-existing comorbidities.

Most children with COVID-19 have a milder clinical course,^2 20^ with the proportion of asymptomatic cases ranging from 15% to 42%^21 22^ and with recovery among symptomatic cases occurring within 2 weeks after disease onset.^2 23 24^ Aligned with the evidence, we found a proportion of 40% asymptomatic cases among children who tested positive. However, a significant proportion of symptomatic children did not recover within 2 weeks, and some experienced delayed resolution of symptoms, taking more than 33 days to recover (9.5%). This indicator is higher than those reported in other studies. In the UK, data analyses from 2020 to 2021 indicated a proportion of symptomatic cases after 28 days of 4.1% for those ≤17 years^25^ and 3.1% for the 5–11 years age group and 5.1% for the 12–17 years age group.^24^ However, these estimates were from the period before the Omicron variant predominated.

In our study, children who tested positive from 2021 onwards seemed to take slightly longer to recover from the acute phase. Large studies have evaluated the effects of COVID-19 in adults as SARS-CoV-2 has evolved,^26^ but less has been explored regarding the impact of different variants on the disease course in children. A study of school-age children (3–16 years) in a city in China, conducted 2 months after a peak in Omicron infections in the region (December 2022), indicated that 3.4% of COVID-19 cases reported symptoms lasting at least 28 days.^27^ This estimate is very close to what was observed in pre-Omicron studies.^24 25^

Although the incidence of SARS-CoV-2 infection with the Omicron variant has been reported to be higher than with the Delta variant in children, the clinical course of moderate or more severe disease was less frequent with Omicron.^28 29^ In our study, the prolonged recovery period observed among children who tested positive during the Omicron surge may be attributed to the increased prevalence of other respiratory viruses. Brazil experienced an off-season increase in influenza A/H3N2 cases between November 2021 and January 2022, during the late spring and early summer, which coincided with the rise of Omicron.^30 31^ At that time, there was a significant increase in influenza cases in places like Rio de Janeiro and São Paulo, and some cases of coinfection with SARS-CoV-2 were reported.^32^ Of the 45 children who tested positive during the Omicron circulation period in our study, 30 (66.7%) tested positive in January 2022. Therefore, some of them may have taken longer to recover from Omicron because they were still recovering from another infectious illness. Additionally, favelas like Manguinhos are characterised by high population density, poverty and inadequate water and sanitation infrastructure, which are risk factors for respiratory infections and create an environment where diseases can spread easily.^33^

Children with symptomatic COVID-19 often exhibit clinical features similar to other common viral respiratory infections, frequently reporting fever, cough, headache and upper respiratory tract symptoms.^20^,^2224 34^ We did not observe a deviation from this symptomatology in the children from our study; however, it was notable that 5 of the 14 children (35.7%) who took longer to recover presented with only a single symptom. A systematic review indicated that just over 40% of COVID-19 cases among those under 19 years old presented with a non-specific symptom.^35^ Therefore, symptom-based screening is neither sensitive nor specific in detecting SARS-CoV-2 infection in children. Other findings from our study differ from what has been reported elsewhere. For example, the median duration of symptoms (11 days, IQR: 7–16) appears longer than what was reported in children in Europe, where it was around 6 days for those under 12 years old and 7 days for the 12–17 years age group.^23 24^

Although the clinical manifestations of COVID-19 among the children in our study are not distinct from what has already been reported—for example, reports of cough vary between 30.0% and 72% across studies^36^—children from Manguinhos appear to take longer to recover from the acute phase of the disease. In an analysis of data from Brazil’s official health surveillance systems covering 2.8 million Brazilian children and adolescents (<18 years) with laboratory-confirmed symptomatic SARS-CoV-2 infection, the presence of symptoms ranged from 3.4% (diarrhoea) to 50.2% (cough).^37^ In another study, conducted in the context of a private general hospital in São Paulo serving a high socioeconomic status population, among 1303 episodes of SARS-CoV-2 infection in individuals under 20 years of age who sought care at the emergency department, the most common symptoms were fever (45.2%), nasal congestion/runny nose (44.2%), cough (39.4%) and headache (35.3%).^38^ Apparently, children from Manguinhos experienced more symptoms during the acute phase of COVID-19 than the national average and compared with a high socioeconomic status group.

Notably, children from Manguinhos also reported gastrointestinal symptoms more frequently (<12% in other studies).^37^,^39^ Gastrointestinal symptoms related to COVID-19 may be caused, for example, by the ACE2 receptor, present in the epithelial cells of the gastrointestinal tract, which serves as an entry point for SARS-CoV-2; or by the impact of stress and mental health, as the gut–brain axis can mediate gastrointestinal symptoms.^40^ Additionally, the Manguinhos territory has certain environmental factors that may contribute to the gastrointestinal symptoms in children, such as soil and water contamination in the streams that run through the community.^11 41^

Although our study focuses on the recovery from the acute phase of COVID-19 and did not assess asymptomatic cases that may have reported late-onset symptoms, or evaluate, across the entire group of positive cases, new or remitting symptoms over time, 6 of the 14 children who took longer to recover experienced symptoms for more than 90 days. They accounted for 4.05% of the total number of positive and symptomatic children in the acute phase (6/148), but meet the definition of long covid as a chronic condition associated with SARS-CoV-2 infection, persisting for at least 3 months as a continuous, relapsing and remitting, or progressive illness affecting one or more organ systems.^42^

It is important to consider that symptoms may return after an apparent recovery or new symptoms may emerge. A study conducted at a post-COVID-19 referral paediatric clinic in Rome, Italy, with children and adolescents (≤18 years) who had been infected with SARS-CoV-2, most of whom were diagnosed when the Omicron variant was predominant (71.2%), found that 23.2% reported at least one persistent or newly developed symptom 3 months after the initial infection.^43^ In the RECOVER Pediatric Observational Cohort Study, 45% of school-age children (6–11 years) and 39% of infected adolescents (12–17 years) reported having at least one prolonged symptom (present for at least 3 months after infection).^44^ The most common prolonged symptoms were fatigue/daytime sleepiness or low energy, feeling anxious, headache, body/muscle/joint pain, trouble sleeping and difficulty with memory or concentration. In our study, cough, sneezing and nasal congestion/runny nose were the most prevalent symptoms. However, as the focus was not on long covid, other important symptoms were not assessed at the beginning of the cohort. It is also important to consider that these symptoms may be influenced by the living conditions of these children. Residing in a favela, they are often exposed to inadequate housing, damp environments, mould and air pollution, all of which can have a significant impact on respiratory health.^45^

One-third of symptomatic children during the acute phase of COVID-19 in our study had a pre-existing condition, a proportion higher than the national average among children who had COVID-19 (3%) and higher than those who required hospitalisation (18%).^37^ Although the occurrence of severe COVID-19 in children and adolescents without underlying health conditions is relatively low, the presence of one or more comorbidities is associated with a higher likelihood of a more severe clinical course.^46^ Our cohort was community-based and consisted of mild COVID-19 cases; however, among the children who took longer to recover from symptoms, 35.7% (5/14) had bronchitis/asthma and/or rhinitis. These conditions may be risk factors for long covid in the paediatric population,^43 47^ emphasising the importance of risk stratification in the paediatric management of COVID-19.

Our study has some limitations that should be considered when interpreting the findings. These relate to both the context in which the study was conducted and the specific methodological constraints encountered during its implementation.

First, approximately 70% of the children included in our study tested positive for SARS-CoV-2 by November 2021, and vaccination for those under 18 years old in Brazil began gradually from June 2021, starting with the 12–17 years age group. This explains why over 90% of the children had not started the COVID-19 vaccination regimen when they tested positive. This may have influenced both the reporting of symptoms and the recovery time, as there is evidence of a lower risk of symptomatic SARS-CoV-2 infection among vaccinated children,^48^ as well as an association between vaccination and a shorter duration of symptoms in adults.^49^ In this regard, a limitation of our study is the low proportion of vaccinated children, which prevented proper assessment of potential differences between vaccinated and unvaccinated individuals, particularly in terms of recovery time.

Second, although the fieldworkers were members of the community, the lack of involvement of the children and the broader community represents a weakness of our study. Another important limitation is that the cohort was recruited as part of the routine care provided by a public primary healthcare service, which may have introduced selection bias. This could have led to a higher inclusion of symptomatic children while under-representing those with mild symptoms who did not seek medical care. Additionally, it may have favoured the inclusion of children with distinct health profiles, such as those receiving regular follow-up due to pre-existing health conditions. In this regard, both recovery time and symptom prevalence might overestimate the true experience of children in the community.

Another challenge encountered during the study was the difficulty in conducting home visits according to the study protocol schedule. The main reasons for these missed visits were armed conflicts within the community and family work commitments outside the area. This may have affected our ability to detect acute SARS-CoV-2 infections occurring at the time of the missed visits and may also have contributed to the small number of test-positive children who were symptomatic.

Moreover, a further limitation concerns the lack of comparison between test-positive and test-negative groups. Although the study protocol included more frequent follow-up calls for symptomatic children, maintaining contact was easier for those who tested positive. As a result, the time between follow-up for the test-negative group was longer in the first month after testing, making group comparisons unfeasible. In future studies of this cohort, however, better group comparisons will be possible. As the cohort expanded and the study progressed, follow-up calls became more frequent, regardless of test results or the presence of symptoms. However, the present analysis focused on the original cohort and the early period of the pandemic, when execution and contact were more challenging. Similarly, future studies could assess long covid by investigating whether asymptomatic children in the acute phase later developed symptoms and whether symptomatic children who recovered experienced a recurrence of symptoms over time.

Among the strengths of our study are the prospective design and the continuous monitoring of symptoms through phone calls, which minimised information bias. Another strength is our analytical approach to identifying change points on the curve of symptom persistence probability. This can help distinguish between the acute phase of the disease, where recovery is more likely, and the chronic phase, where the likelihood of becoming asymptomatic is more consistent and lower. Precisely knowing these change points can assist healthcare professionals in identifying children who bear a heavier burden of COVID-19 and require long-term follow-up and care. From a research practice standpoint, understanding these points can help answer whether predictors of short-term and long-term recovery are fundamentally different. In this regard, we suggest that this approach be explored in studies with larger samples of children and adolescents. The size of the group of symptomatic positive children in our study limited comparisons, including tests and CIs, which due to their wide range may not capture the true variability of the estimates.

To the best of our knowledge, this is the first study to examine the recovery time from the acute phase of COVID-19 in children living in a highly socioeconomically disadvantaged community. This is particularly relevant given that many children in marginalised areas of the Global South have not received sufficient attention in previous COVID-19 research. Our findings underscore the importance of considering the specific challenges faced by different populations, as health disparities can manifest not only between countries but also within the same city, reflecting deeply rooted social and economic inequalities.

In favelas and urban communities, understanding the specific territorial dynamics is crucial for developing effective public health interventions and robust surveillance systems. Popular health surveillance can be an ally in these areas, characterised by a participatory health practice that prioritises the involvement of local communities and social movements. This approach promotes a process of prevention, protection and health promotion by identifying and considering the socioenvironmental dynamics related to the health needs experienced in these territories.^50^ Other actions may include health education campaigns, active vaccination outreach by health professionals, including within the school environment, and strengthening community health worker programmes.

However, addressing health challenges in favelas and urban communities requires more than disease-specific interventions. The social determinants of health must also be tackled through public policies and interventions that focus on addressing poverty, local infrastructure and housing conditions, access to clean water and sanitation, and environmental risk factors. Without addressing social inequalities, the burden of infectious diseases and other health threats will continue to disproportionately affect the most vulnerable groups.

As the threat of COVID-19 aligns with other respiratory viruses, there is a need for unified respiratory virus guidance rather than separate policies for COVID-19. Public health efforts should prioritise hygiene, improved indoor air quality, mask use and comprehensive vaccination, leading to better prevention and reduced viral transmission. Investing in these strategies not only has the potential to strengthen preparedness for future outbreaks but also reduce the burden of respiratory diseases, particularly among children in more disadvantaged environments.

## Conclusion

Our study highlights the need for careful attention to preschool-age children, boys, children with pre-existing comorbidities and those who tested positive during the Omicron circulation period. Our analysis showed that nearly 10% of symptomatic children in the acute phase experienced prolonged symptoms lasting more than 33 days, raising concerns about the persistence of symptoms after COVID-19 in paediatrics and suggesting that from 4 weeks after the initial infection it may be possible to identify children requiring specialised medical attention. Addressing the long-term effects of COVID-19 in children, especially in socioeconomically segregated areas, should be a priority for public health strategies.

## Data Availability

Data are available upon reasonable request.
