# Supplementary material for: Duration of COVID-19 symptoms in children: a longitudinal study in a Rio de Janeiro favela, Brazil
Source: BMJ Open. 2025 Jul 6;15(7):e095622. doi: 10.1136/bmjopen-2024-095622 (PMC12230966; doi:10.1136/bmjopen-2024-095622)
Supplement: Supplementary file 1 [file bmjopen-2024-095622.reviewer_comments.pdf]

# PEER REVIEW HISTORY

BMJ Open publishes all reviews undertaken for accepted manuscripts. Reviewers are asked to complete a checklist review form and are provided with free text boxes to elaborate on their assessment. These free text comments are reproduced below.

## ARTICLE DETAILS

### Title (Provisional)

Duration of COVID-19 symptoms in children: a longitudinal study in a Rio de Janeiro favela, Brazil

### Authors

Oliveira, Fernanda Esthefane Garrides; Bastos, Leonardo; de Oliveira, Raquel de Vasconcellos Carvalhães; Santos, Heloísa Ferreira Pinto; Damasceno, Luana Santana; Franco, Luiza Sales; Carvalho, Liege Maria Abreu de; Fuller, Trevon Louis; Guaraldo, Lusiele; Carvalho, Marilia; Brasil, Patrícia

---

## VERSION 1 - REVIEW

---

| | |
|---|---|
| **Reviewer** | **1** |
| **Name** | **Miranda Montoya, Maria Consuelo** |
| **Affiliation** | **Universidad Industrial de Santander, Facultad de Salud** |
| **Date** | **24-Jan-2025** |
| **COI** | **None** |

---

This manuscript addresses an important topic and offers a valuable contribution by analyzing data from a well-characterized cohort of children followed longitudinally. This approach provides crucial insights into the clinical course of COVID-19 in children residing in a resource-limited setting, reflecting the socioeconomic characteristics of many Latin American countries where such data is currently scarce.

To enhance the clarity and impact of the manuscript, I recommend the following:

Consideration of Control Group: Given the limited data on COVID-19 symptom duration in Latin American children, incorporating a control group is crucial. As recommended by WHO, a suitable control group could consist of symptomatic children who tested negative for SARS-CoV-2, matched by age and temporal proximity.

Enhance Methodological Clarity:

While the manuscript refers to the original cohort study (Reference 4), a more detailed description of the methods specific to this analysis is necessary:

• Clearly state that this analysis focuses on symptomatic children with confirmed SARS-CoV-2 infection from the larger cohort since the abstract to the methods.

• Provide a more comprehensive characterization of the study population, including sociodemographic factors (e.g., nutrition, health problems, schooling, vaccination status).

• Define the eligibility criteria for both the original cohort and the subset analyzed in this study.

Clarify Follow-up Procedures:

• Detail the follow-up procedures for participants, including the duration of follow-up (e.g.," 9 months after the positive test", It can be confused with "Home visits were conducted to collect samples on days 1, 14, and 28, followed by every three months in the first year and twice in the second year". :

• Describe the frequency of phone calls for symptom monitoring and whether these were conducted for all participants or only those with suspected infection. For example, were weekly phone calls established from the beginning? For all participants?

• Clarify the timing of home visits and sample collection in relation to symptom onset and laboratory confirmation. e.g: "Home visits were conducted to collect samples on days 1, 14, and 28, followed by every three months in the first year and twice in the second year". Are these days related to the onset of symptoms or test confirmation? Or was that for everyone after entering the cohort?

• Describe the strategies used to minimize loss to follow-up.

• Specify the criteria used to define a symptomatic case (e.g., number of symptoms required, did any of these symptoms described generate a visit for sample collection?).

Maintain Consistency in Terminology:

Use consistent terminology throughout the manuscript, such as consistently referring to the outcome as "time to symptom resolution." Instead to "loss of symptoms".

Some suggestions in the text:

Title: Duration of COVID-19 Symptoms in Children: A Longitudinal Study in a Rio de Janeiro Favela.

Abstract: Design: Prospective cohort study

Methods: This sentence should be moved to results: "A total of 852 families were included in the study and followed for 24 months, with 1,276 children under the age of 14 enrolled" .

Results:

To maintain consistency in the terminology: Lines 16 to 20: Of the 148 children with acute-phase symptoms who were followed up, 134 experienced symptom resolution, while 14

remained symptomatic at the end of the follow-up period. Among the 134 children who became asymptomatic, the mean time to symptom resolution was…

Lines 43 to 57: Incorporate sample sizes (N) when presenting percentages. This will provide readers with a better understanding of the underlying data.

Recommended literature to enhance the Discussion section:

Camporesi A, Morello R, La Rocca A, Zampino G, Vezzulli F, Munblit D, Raffaelli F, Valentini P, Buonsenso D. Characteristics and predictors of Long Covid in children: a 3-year prospective cohort study. EClinicalMedicine. 2024 Sep 5;76:102815. doi: 10.1016/j.eclinm.2024.102815. PMID: 39296584; PMCID: PMC11408803.

Stephenson, T., Pinto Pereira, S.M., Nugawela, M.D. et al. A 24-month National Cohort Study examining long-term effects of COVID-19 in children and young people. Commun Med 4, 255 (2024). https://doi.org/10.1038/s43856-024-00657-x

Manjula D Nugawela, Snehal M Pinto Pereira, Natalia K Rojas, Kelsey McOwat, Ruth Simmons, Emma Dalrymple, Tamsin Ford, Shruti Garg, Dougal Hargreaves, Malcolm G Semple, Laila Xu, Roz Shafran, Terence Stephenson, CLoCk Consortium , Data Resource Profile: the Children and Young People with Long COVID (CLoCk) Study, International Journal of Epidemiology, Volume 53, Issue 1, February 2024, dyad158, https://doi.org/10.1093/ije/dyad158

Wurm J, Uka A, Bernet V, Buettcher M, Giannoni E, Kottanattu L, et al. The changing clinical presentation of COVID-19 in children during the course of the pandemic. Acta Paediatr. 2024; 113: 771–777. https://doi.org/10.1111/apa.17061

---

| | |
|---|---|
| **Reviewer** | **2** |
| **Name** | **Kleinman, Lawrence C.** |
| **Affiliation** | **Rutgers Health, Pediatrics** |
| **Date** | **26-Feb-2025** |
| **COI** | **No competing direct financial interests. I am funded by the US NIH to do work in this area.** |

---

In general, I like this paper very much and think it makes a contribution.

I would like to see favela defined in the paper and briefly described in the abstract (perhaps, imperfectly, as "slum" or Shanty-town" or descriptor more appropriate to Manguinhos).

I think the paper needs some updating and upgrading of its references. Specifically, the US RECOVER study (with which I am affiliated) has findings relevant to this paper, at least some of which, Dysgeusia in the US sample of adolescents, not in the Brazilian sample, for example, should be specifically reflected upon. Other (including non US) references may be

considered as well to place this study better in the disease and mechanistic context. Some potential references include:

• Gross RS, Thaweethai T, Kleinman LC, et al. Characterizing Long COVID in Children and Adolescents. JAMA 2024;332(14):1174–88. This describes dysgeusia in adolescents.

• Rao S, Gross RS, Mohandas S, et al. Postacute Sequelae of SARS-CoV-2 in Children. Pediatrics 2024;153(3):e2023062570.

• Rao S, Lee GM, Razzaghi H, et al. Clinical Features and Burden of Postacute Sequelae of SARS-CoV-2 Infection in Children and Adolescents. JAMA Pediatr 2022;176(10):1000–9.

• Gross RS, Thaweethai T, Rosenzweig EB, et al. Researching COVID to enhance recovery (RECOVER) pediatric study protocol: Rationale, objectives and design. PloS One 2024;19(5):e0285635.

• Other studies include: PMCID: PMC10336045 (Long Covid in Korea), Lancet Microbe 2023; 4: e745–56 (discusses viral persistence), The Pediatric Infectious Disease Journal 41(8):p 663-665, August 2022. (offers a potential mechanism for brain fog), https://doi.org/10.1136/bcr-2020-239108 persistence of virus in tonsils and in a BMJ Journal. Etc

Page 5 line 30. Defining samples here would be helpful

Were allergies assessed as a comorbidity? This would be interesting to know findings if so

P5 Line 53 specify Covid vaccine status

P 6 Was weakness prostration encompassing of what others may term fatigue? If so, please state explicitly, otherwise that is missing and important.

One design issue: Were those who were not symptomatic early ever assessed for later onset of symptoms? I have some not yet published evidence that even asymptomatic acute infections may lead to prolonged symptoms. In other words, symptoms can emerge over time. If not that is a missed opportunity that should be mentioned in the limitations, but I do not see it as invalidating at all those findings that are articulated.

Change point analysis is interesting and appears valid

Lack of patient and public involvement is a weakness.

Calling endpoint “Loss of symptoms” is very awkward. Cessation of symptoms, resolution of symptoms or something similar would be preferable throughout.

P9 L 27ff is off. Most children lost in the first few days, but the median length of time (when $p=0.5$) is 13 days, not the first few. What were the authors trying to say here? Please fix.

The sentence lines 46-55 was awkward and confusing. Please change to a few simple declarative sentences.

Table one needs confidence intervals (maybe p values too, but certainly CI, for the observed deltas.

Some statement about the heterogeneity (if present) across various comorbidities would have been welcome.

Some reflection upon health care and other factors in the favela compared to other Brazilian children and other norms would be appreciated. What else do we know in general about eh health of children in favelas? About Long COVID in other child populations in brazil?

I would like the discussion to have gone a bit further in its analysis of what this study means, regarding both the disease and these children and some suggestions regarding policy implications, health care implications, schooling implications, etc.

---

## VERSION 1 - AUTHOR RESPONSE

**Reviewer #1**

**(1) Consideration of Control Group: Given the limited data on COVID-19 symptom duration in Latin American children, incorporating a control group is crucial. As recommended by WHO, a suitable control group could consist of symptomatic children who tested negative for SARS-CoV-2, matched by age and temporal proximity.**

**Response:** We agree with the reviewer that such a comparison is important. However, we face a limitation in the follow-up of children with negative test results, as contacts within the first month after the test were more frequent for the positive-test group. As a result, we were able to better monitor the recovery time for this group in the early weeks after the test. Over time, with the expansion of the cohort and changes in the monitoring process (e.g., investigating other symptoms), the frequency of contacts improved. However, the group analysed in this study is our original cohort, which reflects the initial challenges we faced when the COVID-19 pandemic reached Brazil. To address this limitation, we have added the following text at the end of the discussion:

*Other limitations of this study include difficulties in conducting home visits according to the study protocol schedule. The main reasons for these missed visits were armed conflicts within the community and family work commitments outside the area. This may have affected our ability to detect acute SARS-CoV-2 infections occurring at the time of the missed visits and may also have contributed to the small number of test-positive children who were symptomatic. Similarly, another limitation concerns the lack of comparison between test-positive and test-negative groups. Although the study protocol included more frequent follow-up calls for symptomatic children, maintaining contact was easier for those who tested positive. As a result, the time between follow-ups for the test-negative group was longer in the first month after testing, making group comparisons unfeasible. In future studies of this cohort, however, better group comparisons will be possible. As the cohort expanded and the study progressed, follow-up calls became more frequent, regardless of test results or the presence of symptoms. However, the present analysis focuses on the original cohort and the early period of the pandemic, when execution and contact were more challenging.*

**(2) While the manuscript refers to the original cohort study (Reference 4), a more detailed description of the methods specific to this analysis is necessary: Clearly state that this analysis focuses on symptomatic children with confirmed SARS-CoV-2 infection from the larger cohort since the abstract to the methods.**

**Response:** We appreciate the reviewer's suggestion and would like to inform you that:
- We have added a sentence to the abstract to address this:
*Setting: This is a community-based cohort of children living in Manguinhos, a favela in Rio de Janeiro. The cohort was followed through home visits and telephone monitoring of symptoms. The analysis focused on symptomatic children with confirmed SARS-CoV-2 infection from this cohort. Recovery time was defined as the interval between the first date with symptoms and the first date without symptoms following a positive SARS-CoV-2 test.*
- Additionally, we have included an explanation at the end of the "Design, Study Population, and Data Collection" section:
*To assess symptom resolution, the eligibility criteria for the present analysis were that the child had a confirmed SARS-CoV-2 infection and was symptomatic during the acute phase of COVID-19.*

**(3) Provide a more comprehensive characterization of the study population, including sociodemographic factors (e.g., nutrition, health problems, schooling, vaccination status).**
**Response:** We appreciate the reviewer's suggestion and agree that a more comprehensive characterization of the study population is important. However, there are limitations in the availability of information about the population of Manguinhos, which means we were unable to address all of the suggestions. Nevertheless, based on the comments from both reviewers regarding the need for a better understanding of the community and its population, we have added a section in the *Methods* titled *Study Area*, where we discuss the available information about Manguinhos. The following text has been included in this new section:
*In the 2010 Brazilian Demographic Census, Manguinhos had an estimated population of 36,160 inhabitants, of whom 9,686 were children aged 0 to <14 years (27% of the population).[5] Most households had three or more residents (66.5%)[6], and the population was predominantly Black (64%).[7] The local population has declined in recent years, with the most recent Brazilian Demographic Census (2022) estimating it at 28,855 inhabitants, although detailed age distribution data is not yet available.[8]*
*Manguinhos is characterised by one of the lowest Human Development Index (HDI) scores in Rio de Janeiro, among the lowest levels of per capita household income[9], high rates of urban violence, and environmental health issues such as flooding, sewage discharged into canals, and contamination of water used for consumption.[10, 11] A local survey covering 955 households found that among adults aged 25 years or older, 66% of men and 69% of women had between 0 and 8 years of education, and 22% of young people aged 14 to 19 were engaged in some form of work.[12] Among the households surveyed, 35% had experienced food insecurity in the previous three months, regular rubbish collection occurred in only 48%, 9% of residents had asthma or bronchitis, and among adults aged 30 or older, 36% had hypertension, 9% had diabetes, approximately 20% were smokers, and 12% consumed alcohol three or more times per week.[12]*

**(4) Define the eligibility criteria for both the original cohort and the subset analyzed in this study.**
**Response:** We appreciate the reviewer's suggestion and would like to inform you that we have added information regarding the eligibility criteria for both the original cohort and the subset analyzed in this study in the section "Design, Study Population, and Data Collection", as follows:
*This health centre is part of the Brazilian public health system, responsible for primary health care in the Manguinhos territory. All children who visited the health centre between May 2020 and May 2022, whether for elective consultation, immunisation, emergency care, or*

*accompanying a family member, were eligible to participate in the study, regardless of whether they were symptomatic or not.*
*At enrolment, a questionnaire was administered to the child's parents or guardians to collect data on medical history, clinical and demographic factors, and COVID-19 vaccination status. If the child was symptomatic, an additional clinical questionnaire was completed to record symptoms. The child's home address and the contact details of the guardians were obtained, and this information was used to schedule home visits and include other children residing in the same household. Parents or adult guardians who refused to participate, stated they had no time to participate, or children whose parents or legal guardians were not present at the time of the home visit, preventing consent, were not included in the study.[13]*
Regarding the eligibility for the subset analyzed in this study, we have made the change as already indicated in our response to the reviewer's comment number 2.

**(5) Clarify Follow-up Procedures: Detail the follow-up procedures for participants, including the duration of follow-up (e.g.," 9 months after the positive test", It can be confused with "Home visits were conducted to collect samples on days 1, 14, and 28, followed by every three months in the first year and twice in the second year".**
**Response:** The reviewer is correct in pointing out this issue. Indeed, it was not clear that the cohort was designed for families to remain in follow-up for up to 2 years, but for the present analysis, we closed the follow-up period at nine months due to our focus on recovery from the acute phase of the disease. We have revised several parts of the text to make this clearer:
- At the end of the "Introduction" section, we modified the text to: *To assess the time to resolution of symptoms from the acute phase of SARS-CoV-2 infection, the first nine months of follow-up after the date of the positive test were considered.*
- In the first paragraph of the "Design, Study Population, and Data Collection" section, we updated the text to indicate that follow-up can last for up to 2 years: *The Manguinhos family cohort is a prospective and dynamic cohort study of children and their household contacts, who remain in follow-up in the cohort for up to 2 years.*
- In the first paragraph of the "Data Analysis" section, we added the following text to explain why we only considered the nine-month period after the test: *The follow-up period to assess the children's recovery was defined as nine months after the positive test due to the difficulty in maintaining periodic contact after this period, but also because of the interest in recovery following the initial infection. As this is a paediatric population, after a long period, children may experience symptoms again due to common health issues for their age group.*

**(6) Describe the frequency of phone calls for symptom monitoring and whether these were conducted for all participants or only those with suspected infection. For example, were weekly phone calls established from the beginning? For all participants?**
**Response:** We thank the reviewer for pointing out that this was not clear. We have added a revised text in the *Design, Study Population, and Data Collection* section to clarify the study protocol:
*The study protocol stipulated that home visits (for sample collection) and follow-up phone calls would be carried out regardless of whether the child was symptomatic or had a positive test. Active home visits and phone calls were also strategies for retaining families in the cohort, as well as having fieldworkers who lived in the community to facilitate rapport with participants. Visits (for sample collection) were planned for days 1 (enrolment in the cohort), 14, and 28 days after enrolment, followed by once every three months in the first year and twice in the second year. Phone calls were scheduled to take place within 10 days of enrolment, then between 11-17 days, 28-32 days, 42-45 days, at 2, 3, 4, 6, 9, and 12 months,*

*between 15-18 months, and between 21-24 months. When the child was symptomatic, phone calls became more frequent, occurring at least once a week until symptom resolution. Further details on the cohort can be found in a previous publication.*[13]
Additionally, as we mentioned in our response to the reviewer's comment number 1, we have included limitations related to the execution of the research protocol in the *Discussion* section.

**(7) Clarify the timing of home visits and sample collection in relation to symptom onset and laboratory confirmation. e.g: "Home visits were conducted to collect samples on days 1, 14, and 28, followed by every three months in the first year and twice in the second year". Are these days related to the onset of symptoms or test confirmation? Or was that for everyone after entering the cohort?**
**Response:** We appreciate the reviewer's suggestion. As indicated in our response to to the reviewer's comment number 6, we have updated the text in the *Design, Study Population, and Data Collection* section to clarify the study protocol regarding home visits and phone follow-ups. To further specify, home visits for sample collection were scheduled based on the cohort enrolment date, rather than symptom onset or test confirmation.

**(8) Describe the strategies used to minimize loss to follow-up.**
**Response:** We appreciate the reviewer's suggestion and inform that we have added the following text in the *Design, Study Population, and Data Collection* section: *Active home visits and phone calls were also strategies for retaining families in the cohort, as well as having fieldworkers who lived in the community to facilitate rapport with participants.*

**(9) Specify the criteria used to define a symptomatic case (e.g., number of symptoms required, did any of these symptoms described generate a visit for sample collection?).**
**Response:** We appreciate the reviewer's suggestion. Some modifications have been made to the text to clarify this point:

- We added a sentence at the end of the paragraph listing the symptoms evaluated in the study: *The clinical manifestations evaluated in the children included cough, sneezing, nasal congestion/runny nose, sore throat, fever, chills, weakness/prostration (fatigue), difficulty breathing, headache, abdominal pain, diarrhea, nausea, vomiting, conjunctivitis, body rash, arthralgia, seizures, anosmia, and dysgeusia. If at least one of these symptoms was reported at the time of the interview/follow-up contact, the child was then considered symptomatic.*
- We completed a sentence in the *Data Analysis* section to indicate that the presence of any symptom was sufficient to define the child as symptomatic: *Children symptomatic in the acute phase of the disease were defined as those who presented any evaluated symptom on the test date or within up to 10 days from the test date.*

Additionally, as mentioned in our responses to reviewer questions 6 and 7, we have included further details about the study protocol in the *Design, Study Population, and Data Collection* section.

**(10) Maintain Consistency in Terminology: Use consistent terminology throughout the manuscript, such as consistently referring to the outcome as "time to symptom resolution." Instead to "loss of symptoms".**
**Response:** We appreciate the reviewer's suggestion and confirm that the necessary changes have been made throughout the text, including in the table headings.

**(11) Some suggestions in the text. Title: Duration of COVID-19 Symptoms in Children: A Longitudinal Study in a Rio de Janeiro Favela.**
**Response:** We appreciate the reviewer's suggestion and confirm that it has been implemented.

**(12) Some suggestions in the text. Abstract: Design: Prospective cohort study**
**Response:** We appreciate the reviewer's suggestion and confirm that it has been implemented.

**(13) Methods: This sentence should be moved to results: "A total of 852 families were included in the study and followed for 24 months, with 1,276 children under the age of 14 enrolled" .**
**Response:** We appreciate the reviewer's suggestion and confirm that it has been implemented.

**(14) Results: To maintain consistency in the terminology: Lines 16 to 20: Of the 148 children with acute-phase symptoms who were followed up, 134 experienced symptom resolution, while 14 remained symptomatic at the end of the follow-up period. Among the 134 children who became asymptomatic, the mean time to symptom resolution was…**
**Response:** We appreciate the reviewer's suggestion and confirm that it has been implemented.

**(15) Lines 43 to 57: Incorporate sample sizes (N) when presenting percentages. This will provide readers with a better understanding of the underlying data.**
**Response:** We appreciate the reviewer's suggestion. We have reviewed the entire text and included absolute frequencies and total group sizes whenever percentages were reported.

**(16) Recommended literature to enhance the Discussion section:**

- Camporesi A, Morello R, La Rocca A, Zampino G, Vezzulli F, Munblit D, Raffaelli F, Valentini P, Buonsenso D. Characteristics and predictors of Long Covid in children: a 3-year prospective cohort study. EClinicalMedicine. 2024 Sep 5;76:102815. doi: 10.1016/j.eclinm.2024.102815. PMID: 39296584; PMCID: PMC11408803.
- Stephenson, T., Pinto Pereira, S.M., Nugawela, M.D. et al. A 24-month National Cohort Study examining long-term effects of COVID-19 in children and young people. Commun Med 4, 255 (2024). https://doi.org/10.1038/s43856-024-00657-x
- Manjula D Nugawela, Snehal M Pinto Pereira, Natalia K Rojas, Kelsey McOwat, Ruth Simmons, Emma Dalrymple, Tamsin Ford, Shruti Garg, Dougal Hargreaves, Malcolm G Semple, Laila Xu, Roz Shafran, Terence Stephenson, CLoCk Consortium , Data Resource Profile: the Children and Young People with Long COVID (CLoCk) Study, International Journal of Epidemiology, Volume 53, Issue 1, February 2024, dyad158, https://doi.org/10.1093/ije/dyad158
- Wurm J, Uka A, Bernet V, Buettcher M, Giannoni E, Kottanattu L, et al. The changing clinical presentation of COVID-19 in children during the course of the pandemic. Acta Paediatr. 2024; 113: 771–777. https://doi.org/10.1111/apa.17061

**Response:** We appreciate the suggestions provided by the reviewer. We would like to inform you that we have worked on improving our *Discussion* and have included additional references. Some references are more challenging, as our work focuses on the acute phase rather than the long-term effects. We have included the reference suggested by the reviewer (Camporesi et al., 2024) as well as others recommended by reviewer #2.

<u>**Reviewer #2**</u>

**(1) I would like to see favela defined in the paper and briefly described in the abstract (perhaps, imperfectly, as "slum" or Shanty-town" or descriptor more appropriate to Manguinhos).**

**Response:** We appreciate the reviewer's suggestion and agree that a definition may be helpful for the readers. Based on the recommendations of both reviewers, we decided to create a new section in the *Methods* titled *Study Area*, where we also define what a favela is, as follows: *Manguinhos is a neighbourhood in the North Zone of Rio de Janeiro, also known as the Manguinhos Complex due to the presence of multiple favelas (slums). The Brazilian Institute of Geography and Statistics (IBGE in Portuguese – Instituto Brasileiro de Geografia e Estatística) defines favelas and urban communities as popular territories that have emerged through various strategies used by the population to autonomously and collectively meet their housing needs, given the insufficiency and inadequacy of public policies and investments aimed at ensuring the right to the city.[4] These territories reflect the socio-spatial inequalities of Brazilian urbanisation, characterised by a predominance of households with insecure land tenure, a lack or precarious provision of public services, predominantly self-built housing and infrastructure that often do not conform to public regulations, and/or locations in areas with occupation restrictions or environmental risk.[4]*

We opted not to include the definition of favela in the abstract due to the broadness of the definition and space limitations in the abstract section.

We would also like to inform you that the term "favela" is a politically charged term in our country. The adoption of the term to refer to territories such as Manguinhos has been widely discussed with social movements, the academic community, and various government bodies. Therefore, we would like to maintain the use of the term in our text as it aligns with the wishes of the populations in these areas and with the terminology adopted by official institutions.

**(2) I think the paper needs some updating and upgrading of its references. Specifically, the US RECOVER study (with which I am affiliated) has findings relevant to this paper, at least some of which, Dysgeusia in the US sample of adolescents, not in the Brazilian sample, for example, should be specifically reflected upon. Other (including non US) references may be considered as well to place this study better in the disease and mechanistic context. Some potential references include:**

- Gross RS, Thaweethai T, Kleinman LC, et al. Characterizing Long COVID in Children and Adolescents. JAMA 2024;332(14):1174–88. This describes dysgeusia in adolescents.
- Rao S, Gross RS, Mohandas S, et al. Postacute Sequelae of SARS-CoV-2 in Children. Pediatrics 2024;153(3):e2023062570.
- Rao S, Lee GM, Razzaghi H, et al. Clinical Features and Burden of Postacute Sequelae of SARS-CoV-2 Infection in Children and Adolescents. JAMA Pediatr 2022;176(10):1000–9.
- Gross RS, Thaweethai T, Rosenzweig EB, et al. Researching COVID to enhance recovery (RECOVER) pediatric study protocol: Rationale, objectives and design. PloS One 2024;19(5):e0285635.

- Other studies include: PMCID: PMC10336045 (Long Covid in Korea), Lancet Microbe 2023; 4: e745–56 (discusses viral persistence), The Pediatric Infectious Disease Journal 41(8):p 663-665, August 2022. (offers a potential mechanism for brain fog), https://doi.org/10.1136/bcr-2020-239108 persistence of virus in tonsils and in a BMJ Journal. Etc

**Response:** We appreciate the suggestions provided by the reviewer. We would like to inform you that we have worked on improving our Discussion and have included additional references. Some references are more challenging, as our work focuses on the acute phase rather than the long-term effects and on children from the community. We have included the first reference suggested by the reviewer, as well as another recommended by reviewer #1.

**(3) Page 5 line 30. Defining samples here would be helpful**

**Response:** We inform that the text in the section *Design, Study Population, and Data Collection* underwent an extensive review to address all the reviewers' suggestions, including indicating the number of families approached and retention in the cohort. The text explaining the sample of the current study was moved to the first paragraph of the results section. We believe this improved the flow of the text.

**(4) Were allergies assessed as a comorbidity? This would be interesting to know findings if so**

**Response:** The reviewer's question is valid.

- As stated in the first paragraph of the *Measures* section, the comorbidities investigated were bronchitis/asthma, rhinitis, congenital infections, and other comorbidities. To clarify how this was investigated, we added the following text to the paragraph: *During the recruitment interview, families reported on bronchitis/asthma, rhinitis, congenital infections, and other comorbidities (this was an open-ended question allowing families to report any additional conditions).*

- We also added the following text in the *Results* section for further clarification: *Among the 48 children with pre-existing comorbidities, 17 had rhinitis, 17 had bronchitis/asthma, 5 had both rhinitis and bronchitis/asthma, 4 had one of these conditions along with another condition, and 5 had a single condition different from these, with many of the additional conditions involving some form of disability, such as autism.*

**(5) P5 Line 53 specify Covid vaccine status**

**Response:** We appreciate the reviewer's suggestion and inform that we changed the beginning of the text to: *Vaccination status for COVID-19 at the time of a positive RT-PCR [...].*

**(6) P 6 Was weakness prostration encompassing of what others may term fatigue? If so, please state explicitly, otherwise that is missing and important.**

**Response:** We appreciate the question, and indeed, it is important for COVID-19. The symptom "weakness/prostration" corresponds to fatigue. To clarify this for the readers, we have included the term "fatigue" wherever the symptom appears.

**(7) One design issue: Were those who were not symptomatic early ever assessed for later onset of symptoms? I have some not yet published evidence that even asymptomatic acute infections may lead to prolonged symptoms. In other words, symptoms can emerge over time. If not that is a missed opportunity that should be mentioned in the limitations, but I do not see it as invalidating at all those findings that are articulated.**

**Response:** We agree with the reviewer's comment that it is indeed important to consider that asymptomatic children may develop symptoms later, and that these symptoms may be persistent. In the present study, we did not address this analysis, as our focus was on the recovery of symptomatic children during the acute phase. However, we are working on another investigation regarding long COVID in this community, and preliminary results indicate that around 28% of asymptomatic children in the acute phase report some symptoms in the following months. We hope to conclude this research and submit it soon.
Due to the limitations of the present study, we added the following text at the end of the Discussion section when addressing the limitations: *As the cohort expanded and the study progressed, follow-up calls became more frequent, regardless of test results or the presence of symptoms. However, the present analysis focuses on the original cohort and the early period of the pandemic, when execution and contact were more challenging. Similarly, future studies could assess long COVID by investigating whether asymptomatic children in the acute phase later developed symptoms and whether symptomatic children who recovered experienced a recurrence of symptoms over time.*

**(8) Lack of patient and public involvement is a weakness.**
**Response:** We agree with the reviewer. We have added the following text when addressing the study's limitations in the *Discussion* section: *Our study presents some limitations that should be acknowledged. Although the fieldworkers were members of the community, the lack of involvement of the children and the broader community represents a weakness of our study.*

**(9) Calling endpoint "Loss of symptoms" is very awkward. Cessation of symptoms, resolution of symptoms or something similar would be preferable throughout.**
**Response:** The reviewer is absolutely right in their comment. We would like to inform you that the necessary changes have been made throughout the text. As we mentioned in response to the same comment raised by Reviewer #1 in their comment number 10, we have adopted "symptom resolution" throughout the text.

**(10) P9 L 27ff is off. Most children lost in the first few days, but the median length of time (when p=0.5) is 13 days, not the first few. What were the authors trying to say here? Please fix.**
**Response:** We appreciate the careful reading and have adjusted the sentence to make it more appropriate: *Most children became asymptomatic within the first two weeks after the symptom onset, so that by the 13th day, the probability of symptoms persisting was 0.50.*

**(11) The sentence lines 46-55 was awkward and confusing. Please change to a few simple declarative sentences.**
**Response:** We appreciate the comment and have reviewed the text as suggested: *The group of symptomatic children was primarily composed of male children (80/148 – 54.05%), those aged 6 to <14 years (75/148 – 50.68%), children who were not vaccinated for COVID-19 (138/148 – 93.24%), and those without pre-existing comorbidities (100/148 – 67.57%). Children who tested positive during the Gamma/Delta period were slightly more frequent (59/148 – 39.86%).*

**(12) Table one needs confidence intervals (maybe p values too, but certainly CI, for the observed deltas.**
**Response:** We appreciate the reviewer's observation. We had not initially included confidence intervals due to the sample size of our study, which impacts the precision of the estimates. However, we have followed the recommendation and added confidence intervals

for the proportions presented in Table 1. Additionally, we have acknowledged the limitation for comparisons in the *Discussion* section: *The size of the group of symptomatic positive children in our study limited comparisons, including tests and confidence intervals, which, due to their wide range, may not capture the true variability of the estimates.*

**(13) Some statement about the heterogeneity (if present) across various comorbidities would have been welcome.**
**Response:** We consider this point very important. As mentioned in our response to comment number 4, we have added more details about comorbidities in the Results section. In addition to the changes mentioned in response to comment 4, we have included a new column in Table 3 to indicate the pre-existing conditions of the 14 children who took longer to recover from symptoms. Consequently, we have incorporated this description into the last paragraph of the Results section. Furthermore, we have added a new paragraph in the Discussion section to address these issues: *One-third of symptomatic children during the acute phase of COVID-19 in our study had a pre-existing condition, a proportion higher than the national average among children who had COVID-19 (3%) and higher than that of those who required hospitalisation (18%).[37] Although the occurrence of severe COVID-19 in children and adolescents without underlying health conditions is relatively low, the presence of one or more comorbidities is associated with a higher likelihood of a more severe clinical course.[46] Our cohort was community-based and consisted of mild COVID-19 cases; however, among the children who took longer to recover from symptoms, five (5/14 – 35.7%) had bronchitis/asthma and/or rhinitis. These conditions may be risk factors for long COVID in the paediatric population,[43, 47] emphasizing the importance of risk stratification in the paediatric management of COVID-19.*

**(14) Some reflection upon health care and other factors in the favela compared to other Brazilian children and other norms would be appreciated. What else do we know in general about eh health of children in favelas? About Long COVID in other child populations in brazil?**
**Response:** We appreciate the reviewer's suggestion and would like to inform you that we have revised the Discussion section of our manuscript, incorporated additional references and engaged in further discussions. This has considerably expanded our discussion with new paragraphs.

**(15) I would like the discussion to have gone a bit further in its analysis of what this study means, regarding both the disease and these children and some suggestions regarding policy implications, health care implications, schooling implications, etc.**
**Response:** We understand the reviewer's concern and would like to inform you that we have carefully worked on our discussion to include more points related to the environment where these children reside. Furthermore, we have rephrased the final paragraphs of the Discussion: *To the best of our knowledge, this is the first study to examine the recovery time from the acute phase of COVID-19 in children living in a highly socioeconomically disadvantaged community. This is particularly relevant given that many children in marginalized areas of the Global South have not received sufficient attention in previous COVID-19 research. Our findings underscore the importance of considering the specific challenges faced by different populations, as health disparities can manifest not only between countries but also within the same city, reflecting deeply rooted social and economic inequalities.*
*In favelas and urban communities, understanding the specific territorial dynamics is crucial for developing effective public health interventions and robust surveillance systems. Popular health surveillance can be an ally in these areas, characterised by a participatory health*

*practice that prioritises the involvement of local communities and social movements. This approach promotes a process of prevention, protection, and health promotion by identifying and considering the socio-environmental dynamics related to the health needs experienced in these territories.[50] Other actions may include health education campaigns, active vaccination outreach by health professionals, including within the school environment, and strengthening community health worker programmes.*

*However, addressing health challenges in favelas and urban communities requires more than disease-specific interventions. The social determinants of health must also be tackled through public policies and interventions that focus on addressing poverty, local infrastructure and housing conditions, access to clean water and sanitation, and environmental risk factors. Without addressing social inequalities, the burden of infectious diseases and other health threats will continue to disproportionately affect the most vulnerable groups.*

*As the threat of COVID-19 aligns with other respiratory viruses, there is a need for unified respiratory virus guidance rather than separate policies for COVID-19. Public health efforts should prioritise hygiene, improved indoor air quality, mask use, and comprehensive vaccination, leading to better prevention and reduced viral transmission. Investing in these strategies not only has the potential to strengthen preparedness for future outbreaks but also to reduce the burden of respiratory diseases, particularly among children in more disadvantaged environments.*

---

## VERSION 2 - REVIEW

---

| | |
|---|---|
| **Reviewer** | **1** |
| **Name** | **Miranda Montoya, Maria Consuelo** |
| **Affiliation** | **Universidad Industrial de Santander, Facultad de Salud** |
| **Date** | **21-Apr-2025** |
| **COI** | |

---

The authors have done an excellent job refining the manuscript and addressing most prior suggestions, significantly improving its quality. For this revised version, I have the following further recommendations:

Title: While the title incorporates my previous suggestion, I now suggest adding the country for greater impact and context. The revised title should read: "Duration of COVID-19 symptoms in children: a Longitudinal Study in a Rio de Janeiro Favela, Brazil."

Abstract (Results, line 48): To ensure terminological consistency, I recommend revising "lost symptoms" to "becoming asymptomatic."

Discussion (line 46, opening): To strengthen the opening, I suggest modifying the first sentence for increased impact. Consider changing "In this study we demonstrate..." to "Our findings demonstrate that symptomatic SARS-CoV-2 infection..." or "This study revealed that..."

Discussion (Limitations, page 17, line 9): Regarding the limitations section, I suggest a structural refinement for improved flow. Please revise the introductory paragraph to clearly indicate that several limitations will be discussed and should be considered when interpreting the findings. Subsequently, employing varied transitional phrases for each limitation would enhance the fluidity of this otherwise well-supported section.

---

| | |
|---|---|
| **Reviewer** | **2** |
| **Name** | **Kleinman, Lawrence C.** |
| **Affiliation** | **Rutgers Health, Pediatrics** |
| **Date** | **26-May-2025** |
| **COI** | |

---

The paper is improved from the prior version. There is perhaps a bit of unnecessary speculation towards the end of the paper (eg causes of abdominal symptoms), but all in all the manuscript works.

---

## VERSION 2 - AUTHOR RESPONSE

**Reviewer**

(1) Title: While the title incorporates my previous suggestion, I now suggest adding the country for greater impact and context. The revised title should read: "Duration of COVID-19 symptoms in children: a Longitudinal Study in a Rio de Janeiro Favela, Brazil."
**Response:** We thank the reviewer for the suggestion and have modified the manuscript title accordingly to include the country.

(2) Abstract (Results, line 48): To ensure terminological consistency, I recommend revising "lost symptoms" to "becoming asymptomatic."
**Response:** We appreciate the suggestion and have revised the text to replace “lost symptoms” with *More boys became asymptomatic within the first 15 days.*

(3) Discussion (line 46, opening): To strengthen the opening, I suggest modifying the first sentence for increased impact. Consider changing "In this study we demonstrate..." to "Our findings demonstrate that symptomatic SARS-CoV-2 infection..." or "This study revealed that..."
**Response:** We appreciate the reviewer’s recommendation and have revised the opening sentence of the paragraph as follows: *Our findings demonstrate that symptomatic SARS-CoV-2 infection in children under 14 years of age residing in a favela territory in Rio de Janeiro is typically short-lived, with more than half of the children becoming asymptomatic within 15 days.*

(4) Discussion (Limitations, page 17, line 9): Regarding the limitations section, I suggest a structural refinement for improved flow. Please revise the introductory paragraph to clearly indicate that several limitations will be discussed and should be considered when interpreting the findings. Subsequently, employing varied transitional phrases for each limitation would enhance the fluidity of this otherwise well-supported section.

**Response:** We appreciate the reviewer's suggestion and have added an introductory paragraph to the limitations section: *Our study presents some limitations that should be considered when interpreting the findings. These relate to both the context in which the study was conducted and specific methodological constraints encountered during its implementation.*
Additionally, we have included varied transitional phrases between the paragraphs to improve the overall flow of this section.
